# Extracellular vesicles derived from different tissues attenuate cardiac dysfunction in murine MI models

**DOI:** 10.1186/s13062-023-00429-y

**Published:** 2023-11-17

**Authors:** Xuan Liu, Shanshan Shi, Xuedi Geng, Enhao Wang, Qingshu Meng, Mimi Li, Fang Lin, Xiaoxue Ma, Wei Han, Xiaohui Zhou

**Affiliations:** 1 Research Center for Translational Medicine, Shanghai East Hospital, School of Medicine, Tongji University, Shanghai, 200092 China; 2 Shanghai Heart Failure Research Center, Shanghai East Hospital, School of Medicine, Tongji University, Shanghai, 200092 China; 3 Department of Cardiothoracic Surgery, Shanghai East Hospital, School of Medicine, Tongji University, Shanghai, 200092 China; 4 Department of Pathology, Shanghai East Hospital, School of Medicine, Tongji University, Shanghai, 200092 China; 5 Department of Heart Failure, Shanghai East Hospital, School of Medicine, Tongji University, Shanghai, 200092 China

**Keywords:** Ischemic heart diseases, Tissue derived extracellular vesicles, Myocardial dysfunction, Cardiac protection

## Abstract

**Background:**

Extracellular vesicles (EVs) derived from various cell sources exert cardioprotective effects during cardiac ischemic injury. Our previous study confirmed that EVs derived from ischemic-reperfusion injured heart tissue aggravated cardiac inflammation and dysfunction. However, the role of EVs derived from normal cardiac tissue in myocardial ischemic injury remains elusive.

**Results:**

In the present study, normal heart-derived EVs (cEVs) and kidney-derived EVs (nEVs) were isolated and intramyocardially injected into mice after myocardial infarction (MI). We demonstrated that administration of both cEVs and nEVs significantly improved cardiac function, reduced the scar size, and alleviated inflammatory infiltration into the heart. In addition, cardiomyocyte apoptosis was inhibited, whereas angiogenesis was enhanced in the hearts receiving cEVs or nEVs treatment. Moreover, intramyocardial injection of cEVs displayed much better cardiac protective efficacy than nEVs in murine MI models. RNA-seq and protein-protein interaction (PPI) network analysis revealed the protective mRNA clusters in both cEVs and nEVs. These mRNAs were involved in multiple signaling pathways, which may synergistically orchestrate to prevent the heart from further damage post MI.

**Conclusions:**

Collectively, our results indicated that EVs derived from normal heart tissue may represent a promising strategy for cardiac protection in ischemic heart diseases.

**Supplementary Information:**

The online version contains supplementary material available at 10.1186/s13062-023-00429-y.

## Introduction

As one of the most common types of cardiovascular disease, myocardial infarction (MI) remains a major cause of mortality worldwide [1]. Although prompt reperfusion therapy is the preferred strategy and could avoid immediate cardiomyocyte death, many patients subjected to MI still develop ventricular remodeling and eventually heart failure [2, 3]. At present, a growing number of studies have demonstrated the therapeutic potential of strategies aimed at reducing cardiac ischemia injury, such as inhibiting cardiomyocyte death [4], promoting angiogenesis [5], or modulating inflammatory responses [6]. However, most therapeutic approaches are insufficient for myocardial repair, which highlights the urgency to explore more efficient therapies.

Recent studies suggested that EVs are widely involved in the pathophysiology of cardiac ischemic diseases [7–9], due to their diverse inherent characteristics, mainly including less immunogenicity, lower toxicity, higher biocompatibility, and higher engineering potential [10, 11]. To date, enormous studies confirmed that EVs derived from different types of cells, including mesenchymal stem cells (MSCs) [12, 13], embryonic stem cells (ESCs) [14], induced pluripotent stem cells (iPSCs) [15], dendritic cells [16, 17], and macrophages [18], exert their roles in cardiac protection after MI. However, most of the parent cells are currently cultured in a two-dimensional environment with a single-cell type [19], which inevitably leads to an interaction loss with other cell populations that coexist in the in vivo microenvironment [20]. As a contrast, EVs isolated directly from tissues possess some advantages, including tissue specificity [21], source homogeneity [20], and cargo diversity [22], indicating that tissue-derived EVs may provide a better therapeutic effect in vivo. In addition, our recent study confirmed that EVs derived from ischemia-reperfusion injured heart tissue aggravated cardiac inflammation and dysfunction [23]. However, whether normal heart-derived EVs (cEVs) as well as kidney-derived EVs (nEVs) can protect the heart from ischemic injury remains elusive.

In the present study, we investigated the therapeutic effects of cEVs and nEVs in cardiac repair using a mouse permanent MI model. We compared the therapy effects of two different EV samples regarding cardiac function, cardiomyocyte apoptosis, inflammation infiltration, and angiogenesis in vivo and in vitro. By combining RNA-seq analysis and PPI network analysis, we identified several clusters of cardioprotective mRNAs in cEVs or nEVs and provided new insights into the mechanisms underlying the beneficial effects of cEVs and nEVs in cardiac protection.

## Results

### Characterization of cEVs and nEVs

**Fig. 1:**
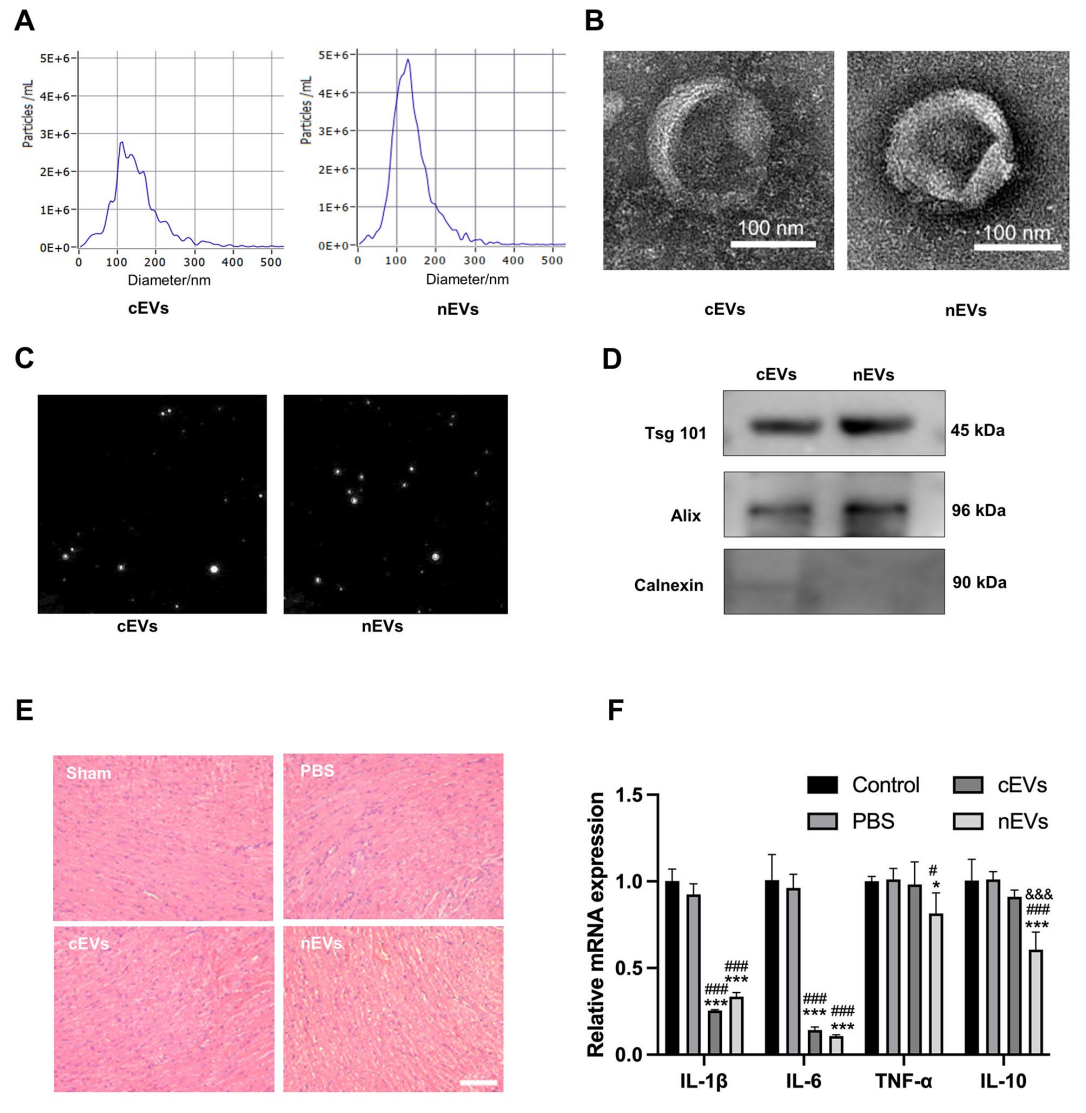
Characterization of cEVs and nEVs. (**A**) NTA analysis of the diameter of cEVs and nEVs. (**B**) Representative TEM micrographs of cEVs and nEVs (scale bar = 100 nm). (**C**) Representative light scattering microscopy (LSM) images of cEVs and nEVs. (**D**) Protein analysis of the EVs markers (Tsg 101, Alix, and Calnexin). (**E**) H&E staining of heart tissue from different groups to assess the biosafety of intramyocardial injection of cEVs and nEVs in vivo. Scar bar:100 μm. (**F**) Relative mRNA expressions of IL-1β, IL-6, TGF-α, and IL-10 in mice hearts among different groups. (*P < 0.05, ***P < 0.001 for MI + cEVs group or MI + nEVs group vs. Sham group; ^#^P < 0.05, ^###^P < 0.001 for MI + cEVs group or MI + nEVs group vs. PBS group; ^&&&^P < 0.001 for MI + nEVs group vs. MI + cEVs group)

The typical biochemical and biophysical characteristics of isolated tissue EVs were identified by TEM, NTA, and western blot, respectively. As shown in Fig. 1A-C, electron microscopy analysis showed that both cEVs and nEVs exhibited a typical cup shape with a diameter of 50–250 nm, which was matched with the size distribution and the brownian motion of particles analyzed by NTA. Besides, these EVs were positive for typical protein markers, including Alix and TSG101, whereas negative for Calnexin, a protein marker of the endoplasmic reticulum (Fig. 1D). Then, we assessed the biosafety of intramyocardial injection of cEVs or nEVs in vivo. We collected the mice hearts and performed H&E staining at day 7 after intramyocardial injection of cEVs or nEVs. There are no obvious pathological changes in mice hearts with cEVs, nEVs, PBS treatment, or sham operation (Fig. 1E). In addition, the mRNA expressions of IL-1β, IL-6, TGF-α, and IL-10 in the heart tissues showed no differences between the sham and PBS group. While, myocardial injection of cEVs or nEVs significantly decreased the expressions of IL-1β and IL-6 in mice heart tissues (Fig. 1F). H&E staining of other major organs, including the liver, spleen, lung, and kidney, also showed no obvious pathological changes among these four groups (Additional File 1: Fig. S1). Taken together, these findings verified the biosafety of intramyocardial injection of cEVs or nEVs in murine MI models.

### Intramyocardial injection of cEVs and nEVs improved cardiac function and reduced scar size post-MI

**Fig. 2:**
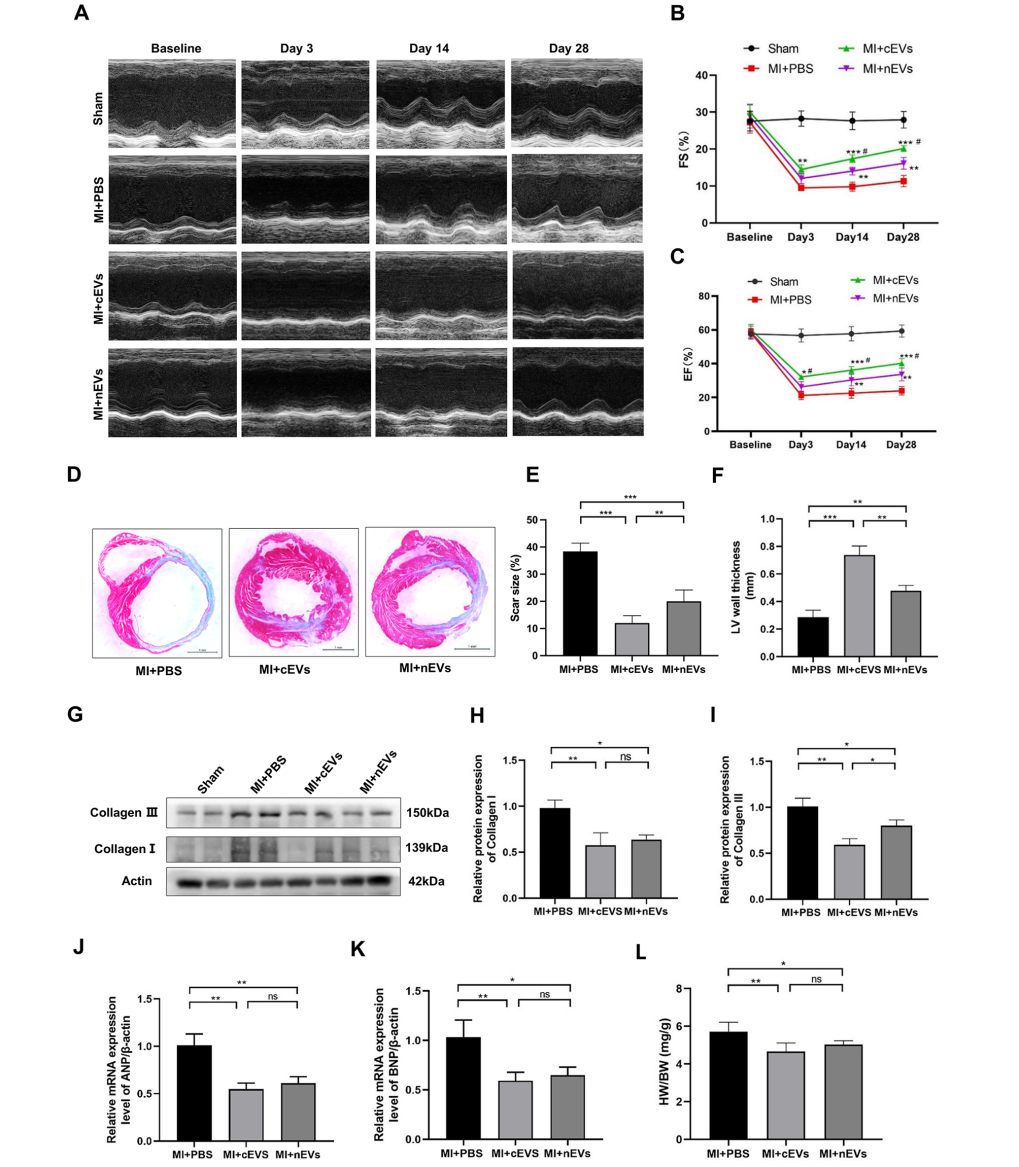
Intramyocardial injection of cEVs and nEVs improved cardiac function and reduced scar size post-MI. Representative M-mode images (**A**) and quantification of EF% (**B**) and FS% (**C**) of Sham, MI + PBS, MI + cEVs, and MI + nEVs groups at baseline, day 3, day 14, and day 28 post-MI (n = 6). Masson’s trichrome staining of mice hearts in different groups at day 28 post-MI (**D**) (scale bar = 1 mm, n = 5), and corresponding statistical analysis (**E**). **F**. Measurement of the left ventricle wall thickness of the mice hearts in different groups at day 28 post operation. **G**-**I**. Protein analysis of collagen **I** and collagen III in mice hearts at day 28 post-MI (n = 4). **J**, **K**. Relative mRNA levels of BNP (**J**) and ANP (**K**) in the MI + PBS group, MI + cEVs group, and MI + nEVs group. L. Analysis of the HW/BW (mg/g) in the MI + PBS group, MI + cEVs group, and MI + nEVs group (n = 6). (*P < 0.05, **P < 0.01, ***P < 0.001 for MI + cEVs group or MI + nEVs group vs. PBS group; ^#^P < 0.05 for MI + nEVs group vs. MI + cEVs group)

To demonstrate the cardiac protection of cEVs and nEVs in vivo, we measured the EF and FS by using echocardiography on pre-operation day (baseline), day 3, day 14, and day 28 post-operation. No significant differences in EF and FS were observed among these four groups at baseline. However, on days 3, 14, and 28 post-operation, both EF and FS were significantly increased in the cEVs and nEVs-treated groups compared to the PBS group. Besides, administration of cEVs showed a better protective effect than nEVs in improving the cardiac function post MI (Fig. 2A-C). Masson’s trichrome staining showed that cEVs administration significantly decreased the scar size of the left ventricle compared with nEVs or PBS-treated groups on day 28 post-MI (Fig. 2D, E). In addition, hearts with cEVs treatment showed much thicker left ventricle wall than that with other two treatments (Fig. 2F). Moreover, we extracted protein in the infarcted area on day 28 post-MI to determine the effect of cEVs or nEVs on cardiac fibrosis. In line with the above findings, both protein expressions of collagen I and collagen III showed a notable decrease in the cEVs and nEVs groups compared with the PBS group, respectively (Fig. 2G-I). Additionally, ANP and BNP expressions in heart tissue was significantly decreased after cEVs or nEVs treatment compared to that treated with PBS (Fig. 2J, K). Meanwhile, the HW/BW indicator in mice receiving cEVs or nEVs presented a significant decline compared to PBS-treated mice (Fig. 2L). Collectively, these results indicated that intramyocardial injection of cEVs and nEVs protected the heart from dysfunction and alleviated cardiac remodeling in mouse MI models, and cEVs displayed much better efficacy than nEVs.

### Both cEVs and nEVs alleviated inflammatory infiltration into the heart after MI

**Fig. 3:**
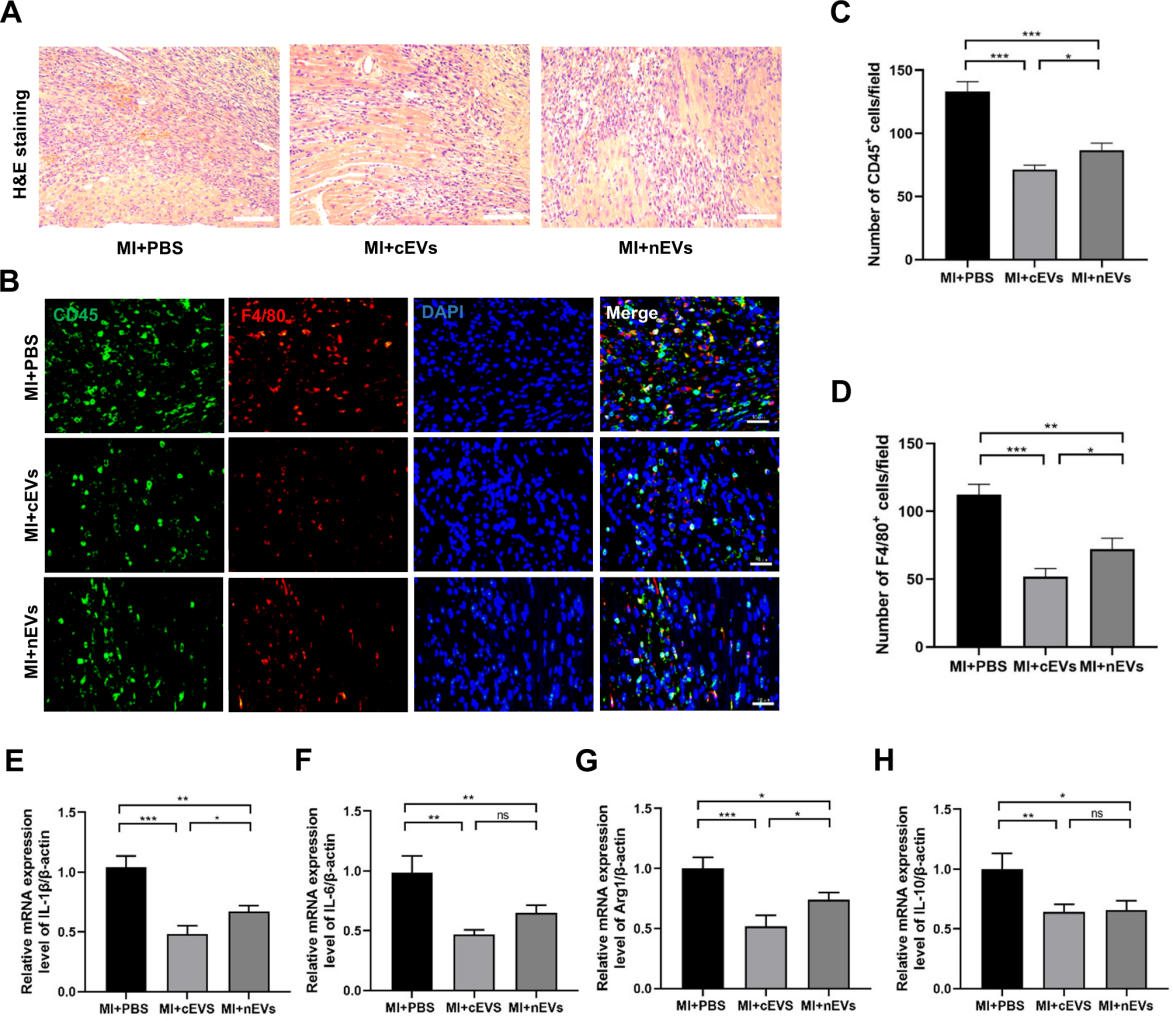
Both cEVs and nEVs alleviated inflammatory infiltration into the heart after MI. (**A**) Representative H&E staining of myocardial sections at 28 days from MI mice treated with PBS, cEVs, or nEVs (scale bar = 100 μm, n = 6). (**B**) Representative immunofluorescence images of myocardial sections stained with anti-CD45 and anti-F4/80 antibodies at 3 days from MI mice treated with PBS, cEVs, or nEVs (scale bar = 50 μm, n = 5–6). (**C**) Statistical analysis of CD45^+^ inflammatory cells (cell counts per field) in the cardiac immunofluorescence images. (**D**) Statistical analysis of F4/80^+^ macrophages (cell counts per field) in the cardiac immunofluorescence images. **E**-**H**. Relative mRNA expressions of IL-1β (**E**), IL-6 (**F**), Arg1 (**G**), and IL-10 (**H**) in the hearts of MI mice treated with PBS, cEVs, or nEVs (n = 3). (*P < 0.05, **P < 0.01, ***P < 0.001, ns, not significant)

Next, we performed H&E staining of the heart tissues to detect the inflammatory infiltration after EVs treatment. As shown by Fig. 3A, both cEVs and nEVs treatment markedly reduced inflammatory infiltration as compared to the PBS group. Then, considering that macrophage infiltration into ischemic tissue and polarization from the pro-inflammatory M1 phenotype towards the anti-inflammatory M2 phenotype are critical to tissue repair [24], we collected the hearts on day 3 post-operation for further analysis. As shown in Fig. 3B-D, cEVs or nEVs treatment significantly downregulated the numbers of CD45^+^ cells and F4/80^+^ macrophages in the ischemic zone compared with the PBS group. Moreover, cEVs treatment further decreased the infiltration of macrophages into the infarcted heart compared with the heart receiving nEVs treatment. To further clarify the role of EVs on macrophage polarization in ischemic myocardium, the M1 polarization-related genes, including IL-1β and IL-6, as well as the M2 polarization-related genes, like Arg-1 and IL-10, were analyzed respectively. As depicted in Fig. 3E and F, the addition of both cEVs and nEVs suppressed the expressions of IL-1β and IL-6 in the heart compared with the PBS group. Besides, the expressions of M2 polarization genes Arg-1 and IL-10 were also decreased in the cEVs group and nEVs group as compared to the PBS group (Fig. 3G, H). Moreover, the expressions of IL-1β and Arg-1 decreased more obviously in cEVs-treated hearts than that receiving nEVs treatment, indicating that cEVs treatment showed better efficiency in modulating the cardiac inflammatory response post infarction.

### Administration of both cEVs and nEVs alleviated cardiomyocytes apoptosis after MI

**Fig. 4:**
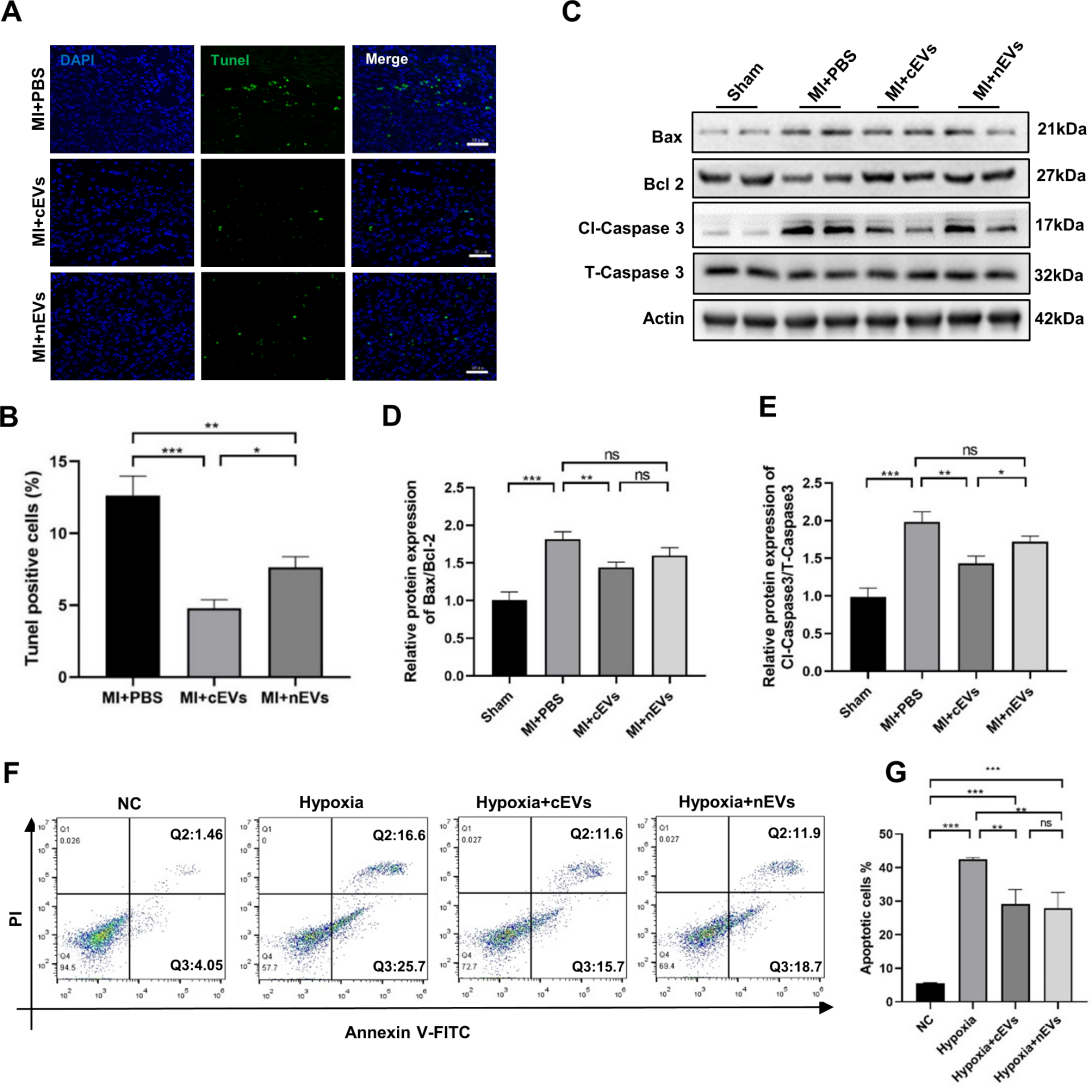
Administration of both cEVs and nEVs alleviated cardiomyocytes apoptosis after MI. (**A**) Representative images of Tunel staining in myocardial sections at 3 days from MI mice treated with PBS, cEVs, or nEVs (scale bar = 50 μm, n = 6). (**B**) Analysis of Tunel positive cells (cell counts per field) in the cardiac immunofluorescence images. **C**-**E**. Protein analysis of Bax, Bcl-2, Cleaved-Caspase 3, and Total-Caspase 3 in mice treated with PBS, cEVs, or nEVs (n = 3). **F**, **G**. Apoptosis rate of cardiomyocytes with different treatments detected by flow cytometry (**F**) and representative analysis (**G**) (n = 4). (*P < 0.05, **P < 0.01, ***P < 0.001, ns, not significant)

Given the fact that ischemia-induced cardiomyocyte apoptosis was critically related to cardiac dysfunction, we then performed Tunel staining of heart sections on day 3 post-operation. As shown in Fig. 4A, B, compared to mice with PBS treatment, myocardial injection of both cEVs and nEVs decreased the number of Tunel positive cells, respectively. Moreover, the number of apoptotic cells in cEVs-treated mice was much less than that in the nEVs-treated group (Fig. 4B). Besides, administration of cEVs decreased both the relative protein expression of Bax/Bcl-2 and the ratio of Cleaved-Caspase 3/total Caspase 3 in mice hearts compared to that receiving PBS treatment (Fig. 4C-E). Moreover, the in vitro experiments demonstrated that addition of both cEVs and nEVs inhibited hypoxia-induced apoptosis in cardiomyocytes (Fig. 4F, G), respectively, which further confirmed the protective role of cEVs and nEVs in preventing cardiomyocyte apoptosis in the setting of MI.

### cEVs and nEVs treatment promoted angiogenesis after MI

**Fig. 5:**
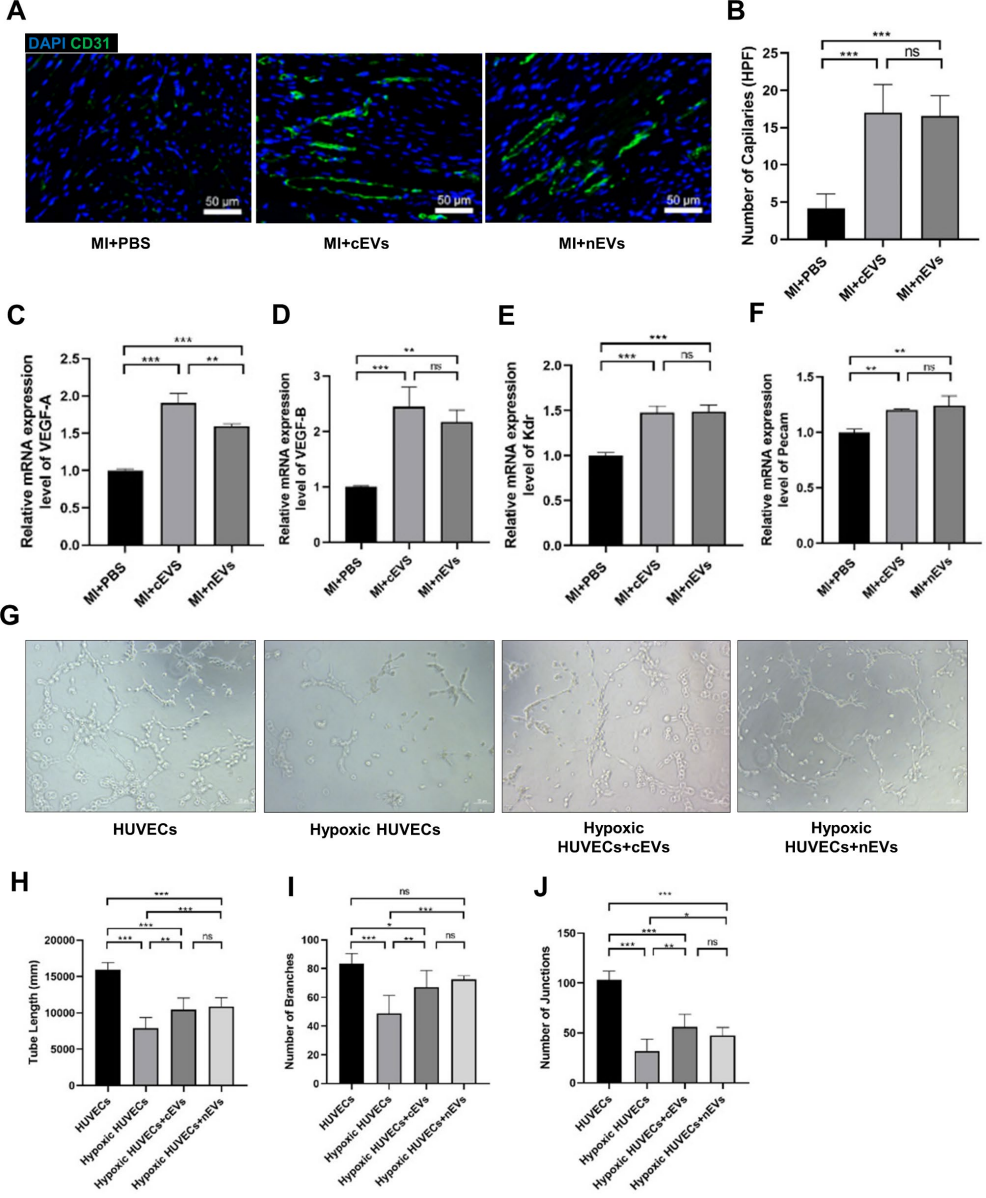
cEVs and nEVs treatment promoted angiogenesis after MI. (**A**) Representative images of CD31 staining in myocardial sections at 28 days from MI mice treated with PBS, cEVs, or nEVs (scale bar = 50 μm, n = 6). (**B**) Quantitative myocardial capillary density in mice hearts at day 28 post-MI. **C**-**F**. Relative mRNA expressions of VEGF-A (**C**), VEGF-B (**D**), Kdr (**E**), and Pecam (**F**) in PBS, cEVs, or nEVs-treated mice hearts at day 28 after MI (n = 3). **G**. Representative images showing the HUVEC-formed tube structure in different groups (scale bar = 200 μm, n = 5). **H**-**J**. Analysis of total tube length (**H**), number of branches (**I**), and number of junctions (**J**) formed by HUVECs in different groups. (*P < 0.05, **P < 0.01, ***P < 0.001, ns, not significant)

Since wound healing after MI involves an endogenous angiogenic responses, we thereby performed CD31 staining, an endothelial cell marker, to detect angiogenesis in the infarction area at 28 days after MI. As shown in Fig. 5A, B, the number of capillaries was significantly higher in the cEVs and nEVs treatment groups than that in the PBS group, whereas no differences were found between the cEVs and nEVs group. Besides, the mRNA expressions of angiogenesis factors in the infarction area, including VEGF-A, VEGF-B, Kdr, and Pecam, were remarkably increased in both cEVs and nEVs treatment groups as compared to the PBS group. In particular, VEGF-A level was much higher in the cEVs group than that in the nEVs group (Fig. 5C-F). To better determine the effect of cEVs or nEVs on angiogenesis, a tube formation assay was conducted in vitro. Fig. 5G-J showed that the tube formation ability of HUVECs was significantly enhanced with cEVs or nEVs treatment, as indicated by the increased total tube length, number of branches, and junctions compared to the control hypoxia group. However, no differences were detected between the cEVs group and the nEVs group. Collectively, these results showed that administration of both cEVs and nEVs can enhance angiogenic response, which contributed to the rebuilding of the blood vessel network and further promoting cardiac healing in the ischemic myocardium.

### RNA-seq analysis of mRNA profiling in cEVs and nEVs

**Fig. 6:**
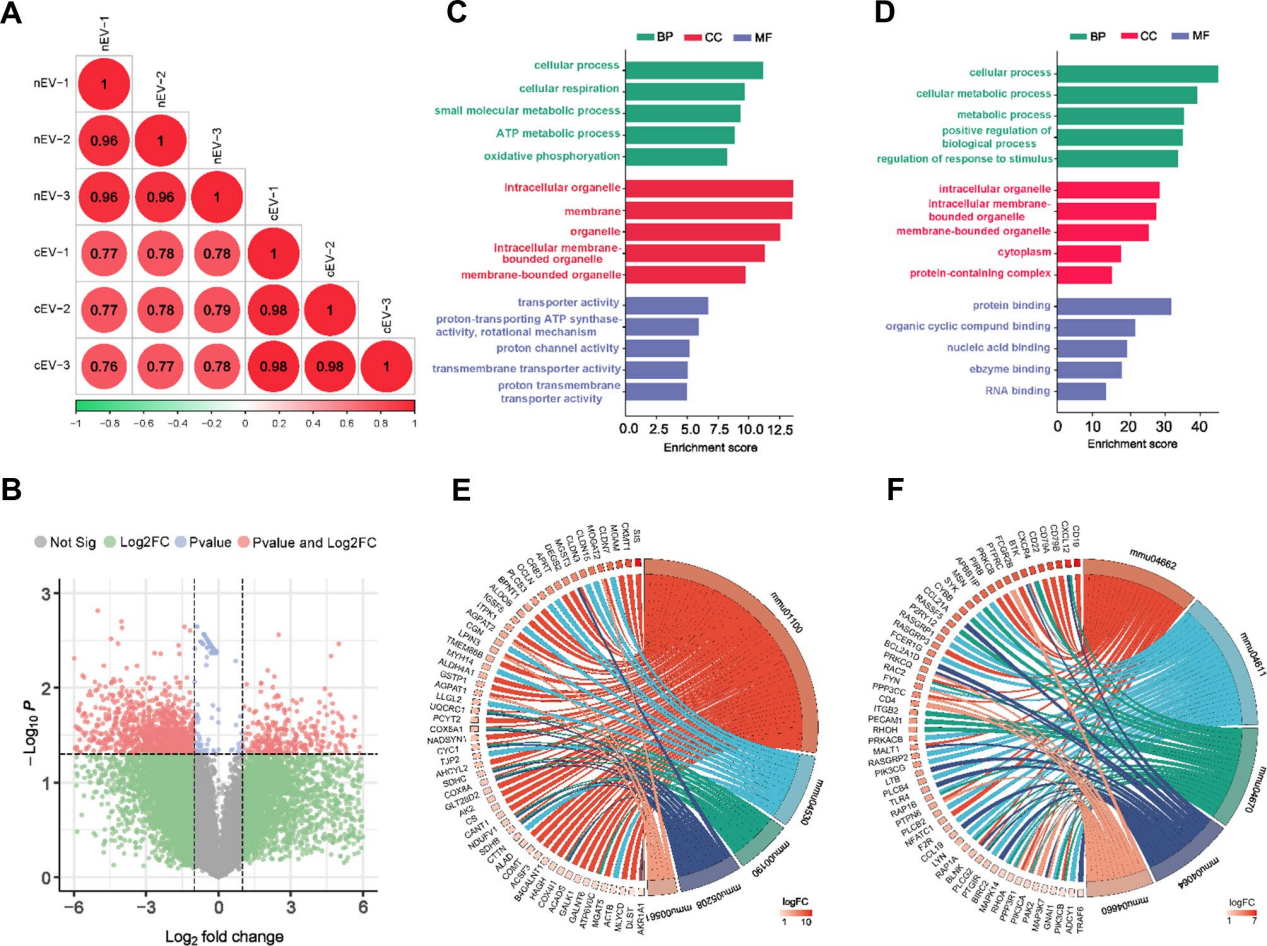
RNA-seq analysis of mRNA profiling in cEVs and nEVs. (**A**) Pearson’s correlation analysis showing a high degree of similarity between the mRNA expression profiles for the cEVs (n = 3) and nEVs (n = 3) samples. (**B**) Volcano plot showing differently expressed mRNAs between cEVs (n = 3) and nEVs (n = 3) samples. Red dot represented differently expressed mRNAs with P < 0.05 and |Log2FC|>1, green dot represented mRNAs with P > 0.05 and |Log2FC|>1, blue dot represented mRNAs with P < 0.05 and |Log2FC|<1, grey dot represented mRNAs with not significant. **C**, **D**. Gene Ontology (GO) analysis of the overexpressed mRNAs in cEVs (**C**) or nEVs (**D**), including biological process (BP), cellular component (CC), and molecular function (MF). **E**, **F**. KEGG analysis of the overexpressed mRNAs in cEVs (**E**) or nEVs (**F**). NOTEs: mmu01100: metabolic pathways; mmu04530: tight junction; mmu00190: oxidative phosphorylation; mmu05208: chemical carcinogenesis - reactive oxygen species; mmu00561: glycerolipid metabolism; mmu04662: B cell receptor signaling pathway; mmu04611: platelet activation; mmu04670: leukocyte transendothelial migration; mmu04064: NF-kappa B signaling pathway; mmu04660: T cell receptor signaling pathway

EVs primarily exert their roles through transferring functional molecules into target cells. In order to characterize the mechanisms of the cardioprotective effects mediated by cEVs and nEVs, we analyzed their mRNA profiling using RNA-seq analysis. Strong positive correlations were found not only between the sample replicates but also between the cEVs and nEVs (Fig. 6A), suggesting the consistency of these EVs samples. Overall, more than 20,000 mRNAs were detected in both cEVs and nEVs (Additional File 1: Fig. S2). After excluding mRNAs with the same expression, we identified 316 and 1043 highly expressed mRNAs in cEVs and nEVs, respectively. (Fig. 6B). Subsequently, we used gene ontology (GO) enrichment analysis to characterize the biological process (BP), cellular compartments (CC), and molecular function (MF) of different expressed mRNAs in cEVs and nEVs. The highly expressed mRNAs in cEVs mainly participated in cellular respiration (BP), the ATP metabolic process (BP), the intracellular organelle (CC), transporter activity (MF), and proton channel activity (MF) (Fig. 6C). Similarly, highly expressed mRNAs in nEVs are mostly involved in the cellular metabolic process (BP), regulation of response to stimulus (BP), intracellular organelle (CC), cytoplasm (CC), protein binding (MF), and nucleic binding (MF) (Fig. 6D). In addition, the KEGG enrichment chord diagram revealed that the top enriched pathways of highly expressed mRNAs in cEVs were involved in metabolic pathways (mmu01100), tight junction (mmu04530), oxidative phosphorylation (mmu00190), chemical carcinogenesis-reactive oxygen species (mmu05208), and glycerolipid metabolism (mmu00561) (Fig. 6E). As a contrast, highly expressed mRNAs in nEVs were involved in the B cell receptor signaling pathway (mmu04662), platelet activation (mmu04611), leukocyte transendothelial migration (mmu04670), NF-kappa B signaling pathway (mmu04064), and T cell receptor signaling pathway (mmu04660) (Fig. 6F). Obviously, GO BP enrichment analysis showed that the highly expressed mRNA in both cEVs and nEVs was associated with cell metabolism, whereas CC, MF, and KEGG enrichment analysis showed significant differences between the mRNAs in cEVs and nEVs. Taken together, these results suggested the possible mechanisms, such as cellular energy metabolism, through which cEVs or nEVs may protect the heart from ischemic injury in murine MI models.

### Identification of protective mRNAs in cEVs and nEVs

**Fig. 7:**
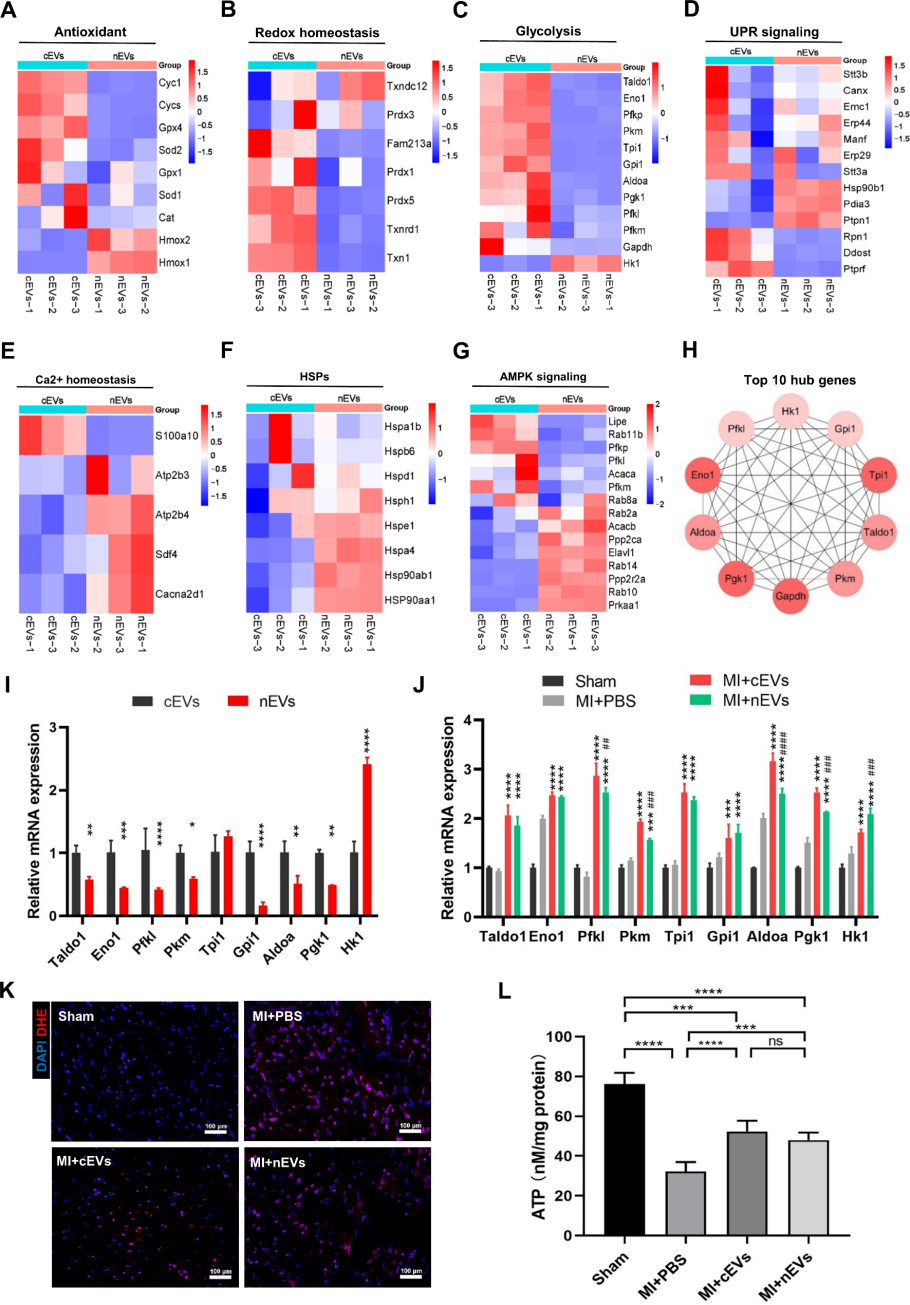
Identification of protective mRNAs in cEVs and nEVs. (**A**-**G**). Heat map illustrating the log2 mass abundance values for the mRNA expression enriched in the cEVs and nEVs involved in cardioprotection pathological processes, including antioxidant (**A**), redox homeostasis (**B**), glycolysis (**C**), unfolded protein response (UPR) signaling pathway (**D**), Ca^2+^ homeostasis (**E**), heat shock proteins (HSPs) (**F**), and AMPK signaling (**G**). **H**. PPI network showing the top 10 hub genes involved in the above cardioprotection pathological processes. **I**. Relative expression of hub top 9 genes (Gapdh was excluded) in cEVs and nEVs. **J**. Relative expression of the top 9 hub genes (Gapdh was excluded) in mice hearts with different treatments 1 day after MI (n = 4). **K**. ROS production in mice hearts of different groups 1 day after MI was indicated by DHE staining (scale bar = 50 μm, n = 4). **L**. ATP content in mice hearts of different groups 1 day after MI (n = 5). (****P < 0.001, ***P < 0.01 for MI + cEVs group or MI + nEVs group vs. MI *+* PBS group; ^###^P < 0.01, ^##^P < 0.05 for MI + nEVs group vs. MI + cEVs group)

Previous studies revealed that upon infarction, multiple signaling pathways can be activated and involved in cardiac-protection. These pathways mainly include antioxidants, redox homeostasis, glycolysis, the unfolded protein response (UPR) signaling pathway, Ca^2+^ homeostasis, heat shock proteins (HSPs), and AMPK signaling [25]. Particularly, mRNAs related to the above protein families and signaling pathways were selected and compared between cEVs and nEVs. As shown in Fig. 7A-C, mRNAs related to antioxidant, redox homeostasis, and glycolysis were highly expressed in cEVs as compared to nEVs, whereas mRNAs related to the UPR signaling pathway (Fig. 7D), Ca^2+^ homeostasis (Fig. 7E), and HSPs (Fig. 7F) were highly expressed in nEVs compared to cEVs. Meanwhile, it seems that mRNAs related to AMPK signaling presented no differences between the two EVs groups. Then, the PPI network analysis was applied to better illustrate the interactions between these protective mRNAs. Although the above-selected 63 mRNAs are grouped into seven different clusters, a high degree of connectivity among the above mRNAs was identified (Additional File 1: Fig. S3), indicating the synergistic activity of the different mRNA clusters in cEVs or nEVs to exert cardioprotective effects. Moreover, the top 10 genes with the highest degree scores in the PPI network were selected as hub genes (Fig. 7H), including Gapdh, phosphoglycerate kinase 1 (Pgk1), aldolase A (Aldoa), enolase 1 (Eno1), phosphofructokinase, liver type (Pfkl), hexokinase 1 (Hk1), glucose-6-phosphate isomerase 1 (Gpi1), triosephosphate isomerase 1 (Tpi1), transaldolase 1 (Taldo1), and pyruvate kinase M1/2 (Pkm). We then verified the relative expression of the top 9 hub genes in cEVs and nEVs by Q-PCR analysis (Gapdh was excluded as it’s regarded as a house-keeping gene) (Fig. 7I). Of note, most of the top genes were included in glycolysis signaling pathways and highly expressed in cEVs. This result indicated the superiority of cEVs than nEVs in treating ischemic cardiac injury may attribute to their effective regulation of the cell metabolism biological process. Subsequently, we further detected these genes in the damaged myocardium of mice from different groups 1 day after MI operation. Compared with mice receiving PBS treatment, the above genes in mice hearts with the administration of cEVs or nEVs significantly increased at different levels (Fig. 7J). In addition, ROS production was notably decreased in mice receiving cEVs or nEVs administration compared to PBS treatment (Fig. 7K). Moreover, the ATP content in mice hearts tissue was also increased after treatment with cEVs or nEVs, which tended to be higher in cEVs-treated mice (Fig. 7L). Collectively, these findings suggested that both cEVs and nEVs can transport protective genes to the injured myocardium, thereby benefiting the attenuation of cardiac injury. The enhanced cellular glycolysis and increased ATP production, as well as inhibited ROS accumulation may at least in part explain the underlying mechanism.

## Discussion

The discovery of cell-free therapy strategies, such as EVs, has been extensively investigated to maximize the therapeutic benefits of donor cells for optimal treatment outcomes [26–28]. Several studies have evaluated the effects of EVs delivery in preclinical large animal models [29, 30], and even large-scale clinical trials are already underway (NCT03384433, NCT03478410). Compared to cell-derived EVs, tissue-derived EVs exist in the extracellular matrix within the tissue microenvironment, which comprises vesicles shed by various cell types [21, 31], indicating that such EVs are well-established mediators of steady intercellular communication. Recently, several kinds of tissue derived EVs, including tumor-derived EVs [32–34], heart-derived EVs [35], brain-derived EVs [36], and small intestine-derived EVs [20] were reported to play central roles in triggering or mediating their parent organ disease process.

The present study, for the first time, disclosed the protective role of EVs isolated from adult mice normal heart and kidney in ischemic cardiac injury. We found that intramyocardial injection of both cEVs and nEVs leads to a range of protective functions associated with cardiac repair, including alleviated inflammatory infiltration, reduced cardiomyocyte apoptosis and scar formation, and enhanced neovascularization in murine MI models. Recent evidence highlighted the importance of therapy strategies targeting cardiomyocytes and endothelial cells, which were reported to play vital roles in post-infarction remodeling and regeneration [37]. However, it is reasonable to suspect that, compared to therapeutic intervention targeting a single type of cell, strategies simultaneously modulating the functional of cardiomyocytes and non-cardiomyocytes in the injured heart may hold greater therapeutic potential regarding cardiac repair. As the reparative response post-MI is a highly dynamic process, it’s likely that both cardiomyocytes and non-cardiomyocytes in the injured mice hearts can get the therapeutic cargo from these normal tissue-derived EVs being intramyocardial injected. Then, the regional cells communicate with each other through both direct cell-cell interactions and indirect paracrine signaling, thereby contributing to a series of healing events after MI [38, 39].

In this article, specifically, cEVs showed a better therapeutic effect than nEVs. Such a superiority may be explained by the different contents carried by these two EVs samples. Growing evidence suggested that the complicated contents of EVs are highly heterogeneous, mainly depending on the types of cell origin, cellular process, and biological environment [40]. In addition to the differences in their cargo itself, the downstream cascades caused by cargo may vary in different target cells, leading to various pathophysiological changes [41]. Our comprehensive RNA-seq analysis revealed the marked differences in mRNA expression profiles between cEVs and nEVs. Unexpectedly, the different expressed mRNAs between the two EVs only accounted for about 7% of the entire mRNA. This phenomenon may be explained partly by the fact that both the heart and kidney of mice originate from the mesoderm. In addition, these two organs are highly metabolized and involved in maintaining the homeostasis of the cardiovascular system. However, the exact mechanism needs further exploration.

When MI occurs, glycolysis is activated in the heart to produce a small amount of ATP to supply energy to hypoxic cardiomyocytes [42]. Then, a series of reactions immediately happened to protect the heart from further damage, such as the antioxidant response, regulation of Ca^2+^ homeostasis, activation of the UPR signaling pathway and the AMPK signaling pathway, etc. [43–45]. As our results showed, both cEVs and nEVs contained mRNAs involved in the above signaling pathways, and it’s still hard to define which pathological changes play the most important cardiac protective roles. We also identified mRNAs that participated in glycolysis as the crucial clusters and further demonstrated that these mRNAs were upregulated in mice hearts after receiving cEVs or nEVs treatment. Besides, administration of cEVs or nEVs remarkably promoted ATP production and inhibited ROS accumulation in mice hearts after MI operation. These findings indicated an enhanced glycolysis and antioxidant response in the damaged myocardium, which may contribute to alleviate cardiomyocyte death. Meanwhile, mRNAs in cEVs or nEVs are engaged in Ca^2+^ homeostasis and other cardiac protective responses, therefore, it is possible that cEVs or nEVs can participate in cardiac healing in another different manners and mechanisms.

Although we confirmed the beneficial role of normal tissue-derived EVs, including cEVs and nEVs, in alleviating cardiac dysfunction in murine MI models, the underlying molecular basis for cardiac protection warrants in-depth exploration. In addition, the present results found that intramyocardial injection of cEVs displayed much better efficacy than nEVs in murine MI models. Further studies are needed to investigate whether EVs from different tissues have their own specificity in treating different diseases. Importantly, since these EVs are derived from vital organs in the body, for translational therapeutic purposes, further researches are needed to determine whether EVs from healthy allogeneic or heterogeneous organs have the same efficacy. Moreover, possible solutions such as reconstitution of 3D tissue-like structures (organoids) and bioreactor-based culture systems may help address the source of EVs and achieve clinical-scale yields of tissue-derived EVs.

## Conclusion

In summary, this study represents a pioneering contribution in demonstrating the protective effects of EVs derived from normal tissues in the treatment of myocardial ischemia injury. Additionally, our findings elucidate that cEVs exhibit significantly superior therapy effect than nEVs in murine MI models. As such, this novel discovery regarding the advantageous roles of tissue-derived EVs may hold substantial promise for advancing cardiac protection strategies in the context of ischemic heart diseases.

## Methods and materials

### Animals

Wild-type C57BL/6 N mice (male, 6–8 weeks old) were purchased from SLAC Laboratory Animal Co., Ltd. (Shanghai, China) and housed in the specific-pathogen-free (SPF) room with constant temperature (23–24 °C), humidity (55 ± 5%), and light (12 hours light-dark cycle). All the mice had free access to standard mouse chow and tap water. This study and all animal procedures conformed to the Guide for the Care and Use of Laboratory Animals, published by the National Institutes of Health (NIH Publication No. 85 − 23, 1996, revised 2011) and approved by the Institutional Animal Care and Use Committee of Tongji University (Number: TJBB00122101).

### Establishment of mice MI models

Acute myocardial infarction was induced by permanent left anterior descending artery (LAD) ligation as described previously [46]. Briefly, the mice were anesthetized with 2% isoflurane and placed in a supine position, with respiration controlled by a rodent ventilator (Nemi Scientific, Inc., Framingham, MA). Subsequently, a left thoracotomy is performed in the fourth intercostal space of the mice, and an 8 − 0 monofilament nylon suture is passed approximately 2–3 mm below the left atrium for permanent ligation of the LAD. After the operation, EVs derived from mice hearts or kidneys were re-suspended in sterile PBS (2.0 × 10^9^ particles/uL in 50 μL PBS) and intramyocardial injected into the left ventricular wall (border zone) at two different locations (MI + cEVs group or MI + nEVs group), respectively. Mice with 50 μL sterile PBS myocardial injection were set as a control (MI + PBS group). Sham-operated mice underwent the same procedure without coronary artery ligation (Sham group). At the indicated time points, mice were sacrificed by an overdose of anesthesia, and tissues were subsequently harvested for histological analysis. Besides, the ratios of heart weight (HW) to body weight (BW) were also calculated for each mouse on day 28 post-operation.

### Echocardiography analysis

Cardiac function was assessed with a Vevo2100 ultrasound system (Visual Sonics, Canada) at different time points (day 3, day 14, and day 28) after MI surgery. Briefly, mice were mildly anesthetized with 1.25% isoflurane and placed on a platform. The left ventricle internal diameters at end-systole (LVESD) and end-diastole (LVEDD) were measured in two-dimensional long-axis views. The left ventricular ejection fraction (EF) and fraction shortening (FS) were calculated for cardiac function assessment.

### Histological examination

The isolated fresh heart tissues were fixed in 4% paraformaldehyde, embedded in paraffin, and then cut into 5 μm-thick slices for hematoxylin and eosin (H&E) staining and Masson trichrome staining to determine the morphological effects and infarct size. The tissue staining blue were considered to have fibrosis and were calculated as the total infarct circumference divided by the total LV circumference × 100. These data were measured and analyzed by Image J software (National Institutes of Health, USA). For immunofluorescence staining, mouse heart sections were incubated with anti-CD45, anti-F4/80, and anti-CD31 antibody (1:100, Cell Signaling Technology, USA) at 4 °C overnight. After washing with PBS for three times, fluorescein-isothiocyanate-conjugated secondary antibodies (1:1000, Cell Signaling Technology, USA) were incubated at room temperature in the dark for 1 hour. Images were acquired using a fluorescence microscope (Leica, Wetzlar, Germany) and quantified using Image J.

### Myocardial apoptosis detection

The effects of EVs on cardiomyocyte apoptosis were detected by Tunel staining. Briefly, mouse hearts were fixed in 4% paraformaldehyde, embedded in paraffin, and cut into 5 μm-thick sections. Afterwards, an In Situ Apoptosis Detection kit (Yeasen, Shanghai, China) was used to detect cardiomyocyte apoptosis according to the manufacturer’s instructions. Cell nuclei were stained with DAPI (Sigma-Aldrich, USA).

### EVs isolation

EVs were isolated by ultracentrifugation, as previously described [47]. In brief, the heart and kidney were perfused with pre-chilled PBS until free of blood. Then, the heart and kidney were removed and rinsed with PBS, minced with sterile scissors, and digested in 0.1% type II collagenase (Sigma-Aldrich, USA) at 37 °C for 30 min with a shaker speed of 300 rpm. Subsequently, the digested tissue was centrifuged at 300 g for 5 min, 2000 g for 10 min, and 10,000 g for 10 min. Thereafter, the supernatant was placed in an ultracentrifuge (Optima L-100XP Ultracentrifuge, Beckman Coulter) and centrifuged at 110,000 g for 70 min. Finally, the pellet was re-suspended in sterile PBS and stored at -80 °C for further use.

### Nanoparticle tracking analysis (NTA)

Nanoparticle tracking analysis (NTA) was performed to detect the particle size and range distribution of EVs by Nano Sight LM10 instruments (Malvern Instruments, Inc., UK), following the manufacturer’s protocols. Briefly, EVs were diluted with PBS at 10^6^ − 10^9^ particles/mL, and the light scattered by the EVs with laser illumination was captured by a camera. Meanwhile, the movement of EVs under brownian motion was also captured and saved as a video file. The NTA 3.1 software was used for analyzing particles individually from 10 to 2000 nm, and the Strokes-Einstein equation was used to calculate their particle size distribution.

### Transmission electron microscopy (TEM)

For TEM, the fresh-isolated EVs were fixed with 2.5% glutaraldehyde stationary liquid overnight. Then 5–7 μL of EVs suspension solution was loaded on the grid and incubated for 1 min. After removing the excess UA solution from the grid by contacting the grid edge with filter paper, dry it for 10 min at room temperature. EVs samples were placed in an EM grid box for observation by a transmission electron microscope (TEM; Hitachi, HT7700).

### Tube formation assay

To detect the effect of cEVs and nEVs on the tube formation ability of endothelial cells. HUVECs (purchased from ATCC) were cocultured with cEVs or nEVs (both 1.0 × 10^9^ particles/mL) for 12 hours, followed by hypoxia treatment with 1% O_2_ and 5% CO_2_ for another 12 hours at 37 °C. Then, a tube formation assay was used to detect the tube formation ability of HUVECs following different treatments. In brief, 50 μL of the Matrigel (Yeasen, China) was added to each well of 96-well plates and incubated at 37 °C for 30 min. Then, a total of 3 × 10^4^ HUVECs per well in each group were seeded on the Matrigel-coated wells. 6 hours later, an inverted microscope was used to observe the tube formation of HUVECs. Image J was used to analyze the tube formation ability of HUVECs in each group.

### Apoptosis detection assay

Mouse Cardiac Myocytes (MCM) cells (purchased from ATCC) were pretreated with cEVs or nEVs (both 1.0 × 10^9^ particles/mL) for 12 hours, followed by hypoxia treatment with 1% O_2_ and 5% CO_2_ for another 12 hours at 37 °C. Then, MCM cells in different groups were washed in ice-cold PBS and collected for the analysis of apoptosis rate with an Annexin V/PI kit (Yeasen, China) according to the manufacturer’s instructions. Briefly, cells were resuspended in 100 μL binding buffer and incubated with 5 μL Annexin V-FITC and 10 μL PI solution for 30 min at room temperature in the dark. Thereafter, another 400 μL of binding buffer was added to resuspend MCM cells. The apoptosis rate of MCM cells in each group was immediately analyzed by flow cytometry (Beckman, Germany).

### Detection of reactive oxygen species (ROS)

Mice hearts were perfused with ice-cold PBS, quickly embedded in an optimal cutting temperature compound (OCT), and frozen in liquid nitrogen. 5 μm-thick sections of the mouse heart were then stained with dihydroethidium (DHE, Invitrogen) for 30 min in the dark, followed by DAPI (Sigma-Aldrich, USA) staining for 3 min. Images were acquired using a fluorescence microscope (Leica, Wetzlar, Germany).

### Detection of ATP in mice heart

The ATP content in the heart tissue of mice from different groups was determined by an ATP detection assay kit (Beyotime, Shanghai, China) according to the manufacturer’s instructions. Briefly, heart tissue under the LAD ligation site and above the apex of the heart is separated and mixed with lysis buffer. After centrifugation at 12,000 g for 5 min at 4 °C, the supernatant is collected for subsequent determination. A 20 μL sample or standard is then added to 100 μL of ATP assay solution. The concentration of ATP in the tissue was measured via luminescence spectrometry. Bicinchoninic acid assay (BCA) protein estimation kit (Thermofisher Scientific, USA) was used to detect sample protein concentration to normalize the ATP content.

### RNA extraction, library construction and construction of gene expression profiles

Total RNA from heart tissues was extracted using TRIzol reagent (Invitrogen, USA). A high-throughput sequencing service was provided by CloudSeq Biotech (Shanghai, China). In brief, total RNA (1 μg) was used for removing the rRNAs using Ribo-Zero rRNA Removal Kits (Illumina, San Diego, CA, USA) following the manufacturer’s instructions. RNA libraries were constructed by using rRNA-depleted RNAs with the TruSeq Stranded Total RNA Library Prep Kit (Illumina, San Diego, CA, USA) according to the manufacturer’s instructions. Libraries were controlled for quality and quantified using the BioAnalyzer 2100 system (Agilent Technologies, Inc., USA). For RNA sequencing, 10 pM libraries were denatured as single-stranded DNA molecules, captured on Illumina flow cells, amplified in situ as clusters, and finally sequenced for 150 cycles on the Illumina NovaSeq 6000 Sequencer according to the manufacturer’s instructions. Then, guided by the Ensembl gtf gene annotation file, Cuffdiff software (part of Cufflinks) was used to get the gene level FPKM as the expression profiles of mRNA, and fold change and p-value were calculated based on FPKM. Differentially expressed mRNA were identified. GO and Pathway enrichment analysis were performed based on the differentially expressed mRNAs. For detection of mRNA expression, the resulting cDNA was amplified by semi-quantitative RT-PCR using SYBR Green MasterMix (Applied Biosystems; Thermo Fisher Scientific, Inc., USA) with three replicates. β-actin was used as the endogenous control. The 2^–∆∆CT^ method was used to calculate the relative expression of different genes. All the primers used in the study were listed in Additional File 1: Table 1.

### PPI network construction

To explore the association of mRNAs in cEVs and nEVs, we used the PPI biological network through the STRING database (https://string-db.org/). Cytoscape software was used for the visualization of interactions [48]. In addition, CytoHubba was used to rank the gene and find the top 10 hub genes in functional networks [49].

### Western blotting analysis

Heart tissue samples and EVs were homogenized in RIPA buffer containing protease and phosphate inhibitors. Protein quantification was carried out using the BCA protein estimation kit (Thermofisher Scientific, USA) in accordance with the manufacturer’s instructions. The antibodies used in this study were listed as follows: Alix (Abcam, UK), Tsg 101 (Abcam, UK), Calnexin (Abcam, UK), Collagen I (Abclone, China), Collagen III (Abclone, China), Bax (Cell Signaling Technology, Inc. USA), Bcl-2 (Cell Signaling Technology, Inc. USA), Cleaved-Caspase-3 (Cell Signaling Technology, Inc. USA), Caspase-3 (Cell Signaling Technology, Inc. USA), and β-actin (Santa Cruz Biotechnology, USA). Total proteins extracted from tissue samples or EVs were separated by sodium dodecylsulfate polyacrylamide gel electrophoresis (SDS-PAGE) gel and transferred to polyvinylidene difluoride (PVDF) membranes. After blocking with 3% non-fat milk in Tris-buffered saline with 0.1% Tween 20 (TBST) for 1 hour at room temperature, the PVDF membrane was incubated with the primary antibody at 4 °C overnight and the secondary antibody at 37 °C for 1 hour, respectively. Then, specific bands were detected by ECL reagent (Share-Bio, Shanghai) and quantified using Image J software.

### Statistical analysis

Results were presented as mean ± SEM. Data normality was determined by the Shapiro-Wilk test. Data obtained from multiple groups were compared using ANOVA, followed by Bonferroni post hoc analysis. A P-value less than 0.05 was considered significant. GraphPad Prism 8.0 (Graph Pad Prism Software Inc., San Diego, CA, USA) was used for statistical analysis.

### Electronic supplementary material

Below is the link to the electronic supplementary material.

Supplementary Material 1

## Data Availability

The datasets used and analyzed during the current study are available from the corresponding author on reasonable request.

## Abbreviations

cEVs: Heart-derived EVs
DHE: Dihydroethidium
EVs: Extracellular vesicles
MI: Myocardial infarction
nEVs: Kidney-derived EVs
NTA: Nanoparticle tracking analysis
PPI: Protein-protein interaction
ROS: Reactive oxygen species
TEM: Transmission electron microscopy
