# Supplementary material for: Extracellular vesicles derived from different tissues attenuate cardiac dysfunction in murine MI models
Source: Biol Direct. 2023 Nov 17;18:76. doi: 10.1186/s13062-023-00429-y (PMC10655353; doi:10.1186/s13062-023-00429-y)
Supplement: Supplementary file 1 — Supplementary Material 1 [file 13062_2023_429_MOESM1_ESM.docx]

**Supplementary Figure. 1 Biosafety assessment of cEVs and nEVs in vivo**

Representative H&E staining of major organs (Liver, Spleen, Lung and Kidney) in mice 7 days after receiving cEVs or nEVs myocardial injection. (scale bar = 100 μm, n=4).


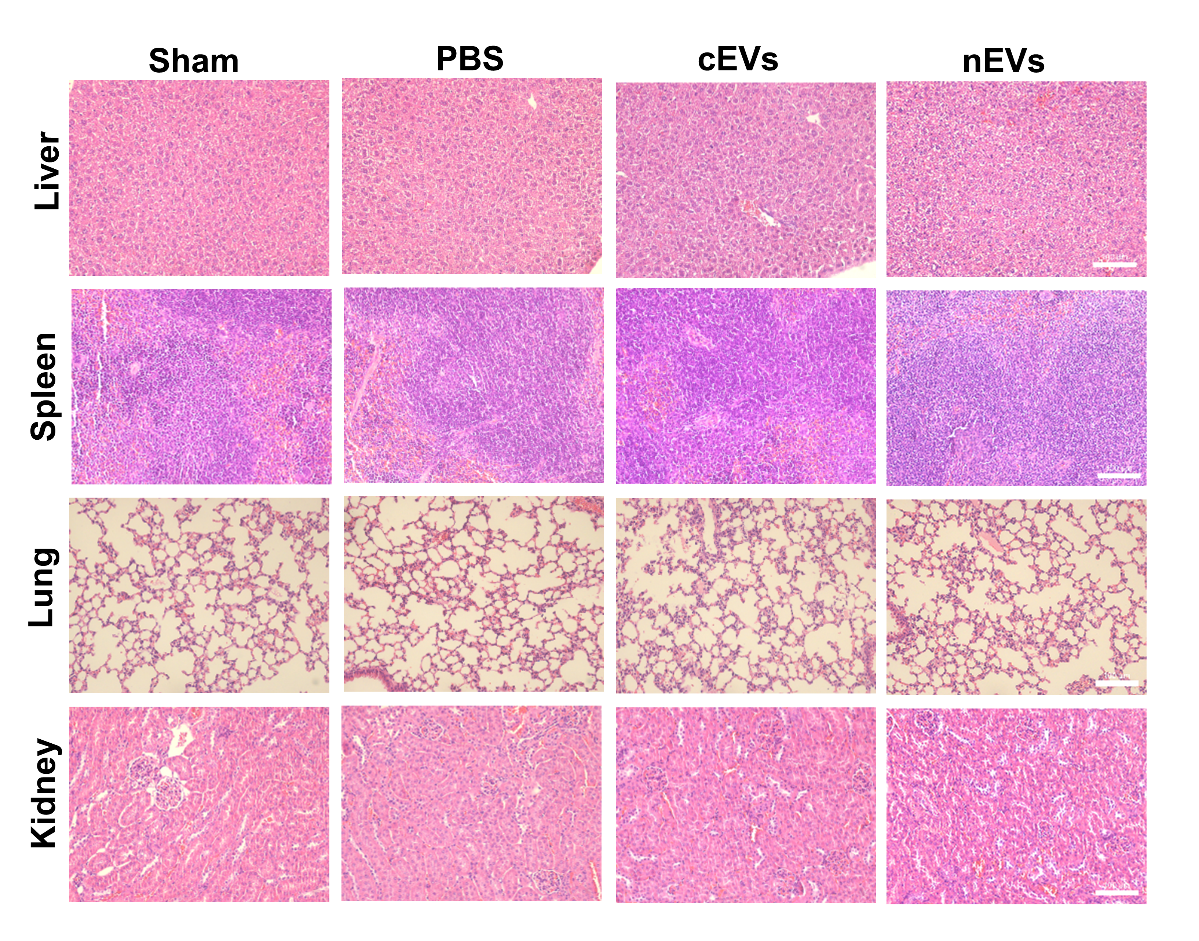


**Supplementary Figure. 2 Heatmap showing different expressed mRNAs in cEVs and nEVs.**

Heatmap showing different expressed mRNAs in cEVs and nEVs, with316 overexpressed mRNAs in cEVs and 1043 overexpressed mRNAs in nEVs.


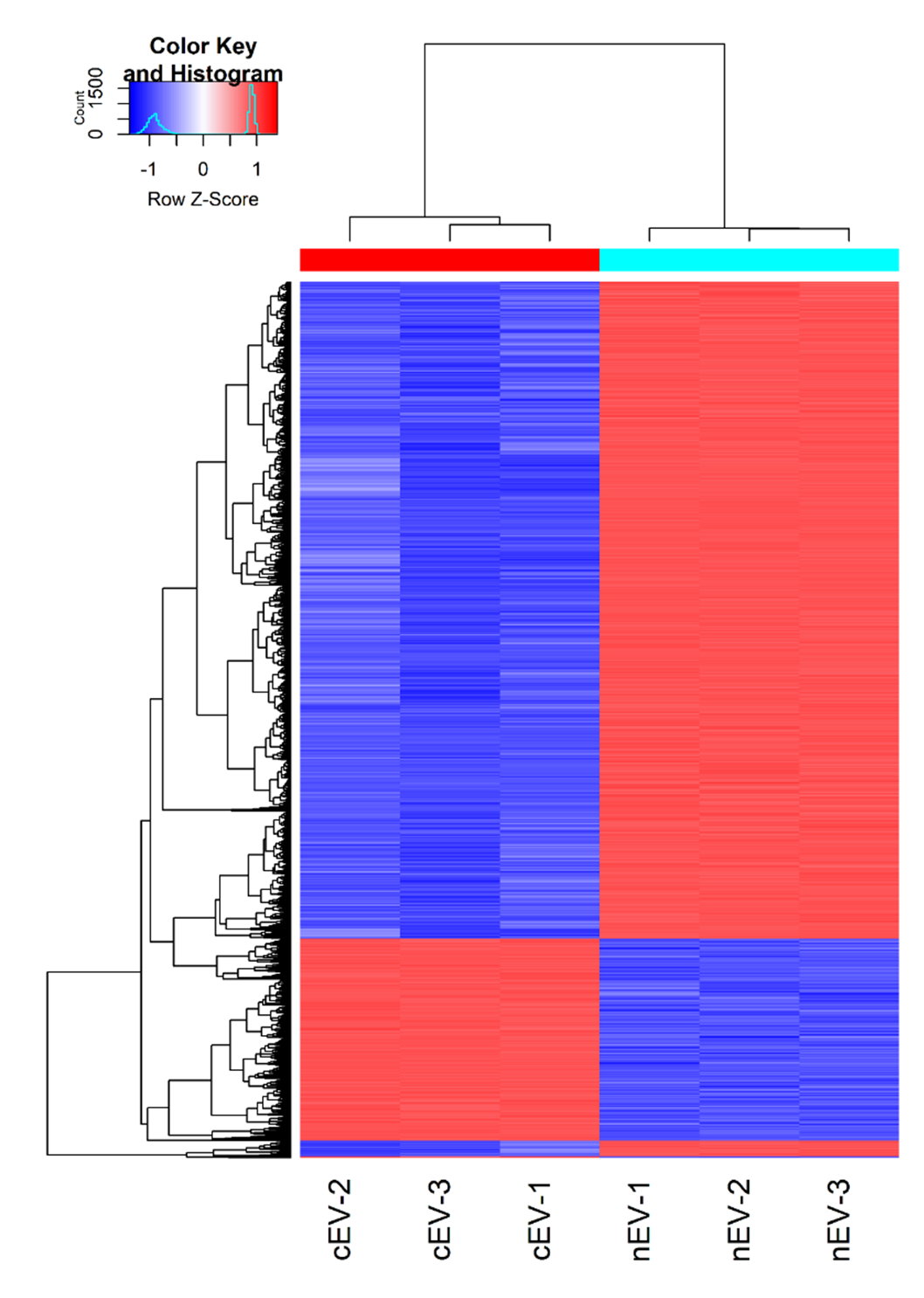


**Supplementary Figure. 3 PPI network among mRNAs involved in different signaling pathways**

PPI network analysis illustrating known interactions between the mRNAs involved in different cardiac protective signaling pathways. The node represents mRNAs, while the edge represents interaction of mRNAs. The higher the degree, the redder of the node and the larger the shape of the node.


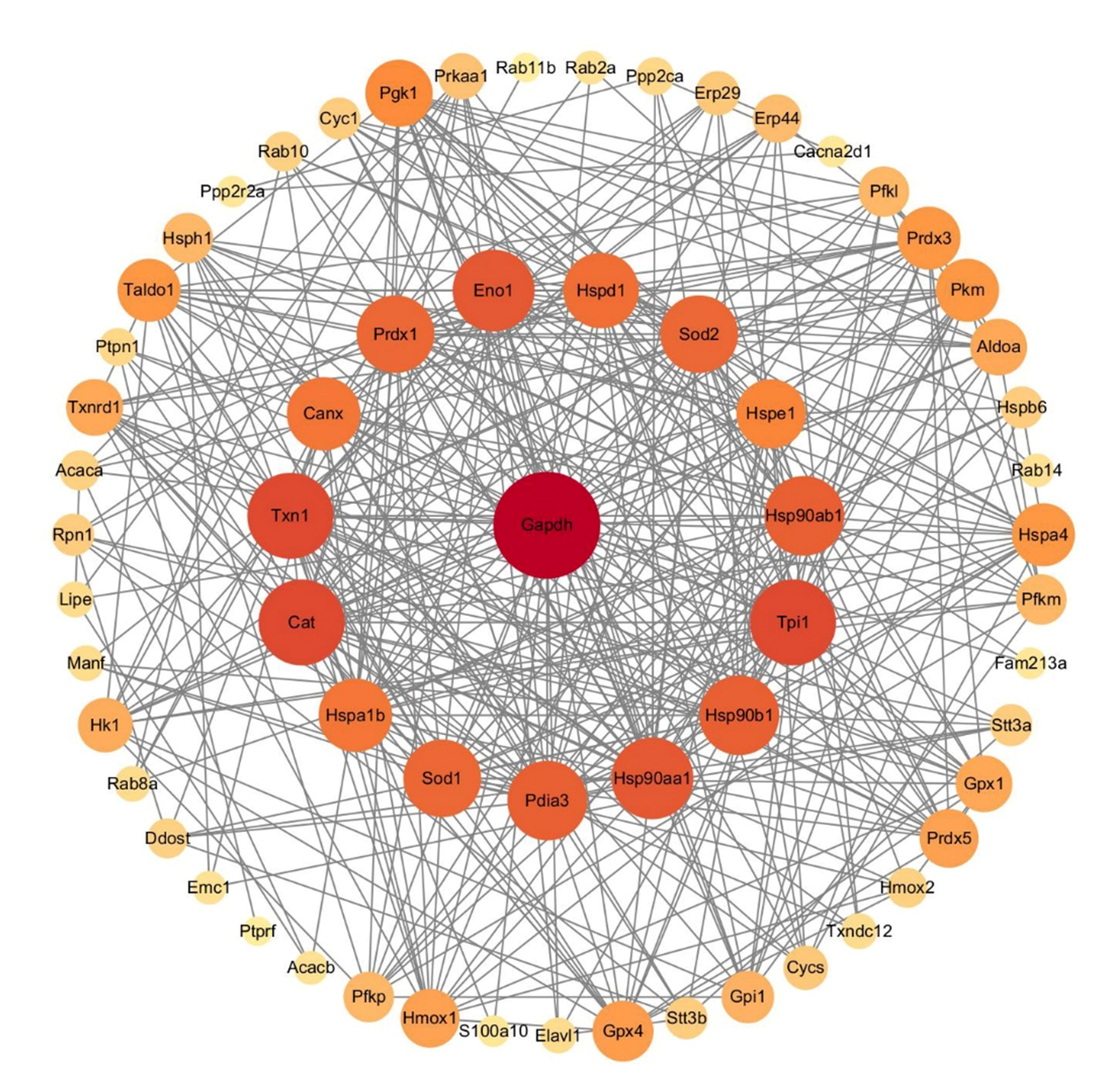


**Supplementary table. 1 Primer sequences used for RT-qPCR in this article.**

| Gene | Forward primer (5′-3′) | Reverse primer (5′-3′) |
| --- | --- | --- |
| GAPDH | ACAGCAACAGGGTGGTGGAC | TTTGAGGGTGCAGCGAACTT |
| IL-1β | GCAACTGTTCCTGAACTCAACT | ATCTTTTGGGGTCCGTCAACT |
| IL-6 | TAGTCCTTCCTACCCCAATTTCC | TTGGTCCTTAGCCACTCCTTC |
| Arg1 | CTCCAAGCCAAAGTCCTTAGAG | GGAGCTGTCATTAGGGACATCA |
| IL-10 | GCTCTTACTGACTGGCATGAG | CGCAGCTCTAGGAGCATGTG |
| ANP | GCTTCCAGGCCATATTGGAG | GGGGGCATGACCTCATCTT |
| BNP | GAGGTCACTCCTATCCTCTGG | GCCATTTCCTCCGACTTTTCTC |
| VEGF-A | GCACATAGAGAGAATGAGCTTCC | CTCCGCTCTGAACAAGGCT |
| VEGF-B | GCCAGACAGGGTTGCCATAC | GGAGTGGGATGGATGATGTCAG |
| Kdr | TTTGGCAAATACAACCCTTCAGA | GCAGAAGATACTGTCACCACC |
| Pecam1 | ACGCTGGTGCTCTATGCAAG | TCAGTTGCTGCCCATTCATCA |
| Tpi1 | CCAAACAATGAGCACTGCCC | GAAACGGCTCAGGAGGGTAC |
| Eno1 | GAGCGAGAAGTCCTGCAACT | CCACCAGGTCTGCGATGAAA |
| Pgk1 | CAAGCTACTGTGGCCTCTGG | GACTTGGTTCCCCTGGCAAA |
| Pkm | CCCCTCCCCTATCCTTTCCA | GTCCTGCATTCCTCCTCCAC |
| Taldo1 | AAATGGCCGTGGAGAAGCTC | CTCAAAGGCAGTGCAGGTCT |
| Aldoa | GCTGTCACTGGGGTCACTTT | TTCTCCTTCTTCCCACCCCA |
| Gpi1 | TGCTCTCCAGTCACGTGTTC | CAGGACCTCTGGAAGAGCAG |
| Hk1 | CTGAAGGGCGAGTGTGAACA | CCTACCACCACCACCATCAC |
| Pfkl | TCATCAGATCCCATTGCAGA | GGAGGGTTCCCTAAATTCCA |
